# PET Evaluation of Microglial Activation in Non-neurodegenerative Brain Diseases

**DOI:** 10.1007/s11910-019-0951-x

**Published:** 2019-05-28

**Authors:** Christine Ghadery, Laura A. Best, Nicola Pavese, Yen Foung Tai, Antonio P. Strafella

**Affiliations:** 1 The Edmond J. Safra Program in Parkinson’s Disease & Movement Disorder Unit, Toronto Western Hospital & Krembil Research Institute, University Health Network; Research Imaging Centre, Campbell Family Mental Health Research Institute, Centre for Addiction and Mental Health, University of Toronto, Toronto, Ontario Canada; 2 Clinical Ageing Research Unit, Newcastle University, Campus for Ageing and Vitality, Westgate Road, Newcastle Upon Tyne, UK; 3 PET centre, University of Aarhus Denmark, Aarhus, Denmark; 4 Imperial College London South Kensington Campus, London, UK

**Keywords:** Neuroinflammation, Microglial activation, TSPO, PET, Neurological diseases, Neuropsychiatric diseases

## Abstract

**Purpose of the Review:**

Microglial cell activation is an important component of neuroinflammation, and it is generally well accepted that chronic microglial activation is indicative of accumulating tissue damage in neurodegenerative conditions, particularly in the earlier stages of disease. Until recently, there has been less focus on the role of neuroinflammation in other forms of neurological and neuropsychiatric conditions. Through this review, we hope to demonstrate the important role TSPO PET imaging has played in illuminating the pivotal role of neuroinflammation and microglial activation underpinning these conditions.

**Recent Findings:**

TSPO is an 18 kDa protein found on the outer membrane of mitochondria and can act as a marker of microglial activation using nuclear imaging. Through the development of radiopharmaceuticals targeting TSPO, researchers have been able to better characterise the spatial-temporal evolution of chronic neurological conditions, ranging from the focal autoimmune reactions seen in multiple sclerosis to the Wallerian degeneration at remote parts of the brain months following acute cerebral infarction.

**Summary:**

Development of novel techniques to investigate neuroinflammation within the central nervous system, for the purposes of diagnosis and therapeutics, has flourished over the past few decades. TSPO has proven itself a robust and sensitive biomarker of microglial activation and neuroimaging affords a minimally invasive technique to characterise neuroinflammatory processes in vivo.

## Introduction

Microglia constitute approximately 5–10% of adult brain cells and are found throughout brain tissue [1••]. They are the resident immune cells of the brain and represent an important effector in the innate immunity of the brain [1••]. Their capacities, including phagocytosis, proliferation and secretion of soluble molecules, mean they are implicated in the neuroinflammatory pathological processes underpinning a number of neurological and neuropsychiatric conditions.

Microglia become “activated” when the integrity of the central nervous system (CNS) is challenged, for example, in the presence of vascular or tissue damage [2••]. Under these circumstances, microglia shift from a sensing state to a reactive state, triggering the release of cytokines, proteinases, complement proteins and reactive oxygen species [3]. The reactive phenotype can then be further subdivided into a pro-inflammatory state (M1) or one characterised by anti-inflammatory reactions (M2) supporting tissue repair, regeneration and coordination of the immune response [2••]. Polarisation of microglial cells is a dynamic, context-specific process depending on the type of stimulus, with temporal integration determining the neuroinflammatory changes seen in the acute versus the chronic setting [4, 5].

Targeted positron emission tomography (PET) imaging of microglia facilitates the in vivo assessment of neuroinflammation through the development of radiopharmaceuticals that target biomarkers of microglial activation. Paramount amongst these is the translocator protein 18-kDa (TSPO), which was previously described as a peripheral benzodiazepine receptor following its identification in through binding studies using ^3^H-diazepam [6]. Under normal circumstances, TSPO is moderately expressed in healthy brain tissue and only minimally expressed in resting microglia. However, with the homeostatic disturbance that inevitably accompanies neuroinflammation, TSPO expression substantially increases, and this is predominantly observed in activated microglia [7]. TSPO upregulation has also been observed in astrocytes, for example following cerebral ischaemia [8], and macrophages that infiltrate the damaged brain due to disruption of the blood-brain barrier and increased vascular permeability secondary to neuroinflammation [9].

The exact role of TSPO, however, remains unclear. It is postulated to be involved in a number of cellular functions ranging from regulation of cell proliferation to cell apoptosis, and its function in the stimulation of microglia is also poorly understood, with most studies suggesting a possible role in reactive oxygen species attenuation [2••, 10, 11]. Despite the lack of functional clarity, TSPO upregulation remains a relevant biomarker, and longitudinal studies utilising TSPO PET have provided insights into the temporal dynamics of neuroinflammation leading to neuronal injury and the progression of chronic disease. Furthermore, TSPO PET allows researchers to determine the efficacy of emerging anti-inflammatory therapies.

Over the past two decades a number of TSPO ligands have emerged, which can be subdivided into first, second and third generation ligands. The most prominent, and still most widely used, is the first generation TSPO ligand ^11^C-PK11195 (PK). PK shows high affinity for binding to TSPO; however, its clinical utility is somewhat limited by the relatively short half-life of carbon-11, requiring an on-site cyclotron, poor signal-to-noise ratio as a consequence of high non-specific binding and low brain bioavailability [12, 13•].

In an attempt to overcome the difficulties with PK in the pursuit of improved image quality, investigators sought to develop superior ligands, including phenoxyarylactemides derivatives (e.g.^11^C-PBR28, ^11^C-DAA1106 and ^18^F-PBR06), pyrazolopyrimidines derivatives (e.g. ^18^F-DPA-714) and imidazopyridine derivatives (e.g. ^11^C-CLINME) [2••]. However, the second-generation ligands have not been without their own drawbacks, and the most significant concerns their sensitivity to the rs6971 polymorphism (Ala147Thr) in the TSPO gene. This affects binding affinity properties causing significant heterogeneity in PET imaging and skewing interpretation of the associated quantitative data. Consequentially, the third-generation radioligands have emerged in an attempt to develop rs6971-insensitive ligands. These TSPO tracers include flutriciclamide (^18^F-GE180) [14••] and ^11^C-ER176 [15]; however, their clinical relevance is still to be determined.

In this review, we will discuss how TSPO PET imaging has been utilised to characterise the neuroinflammatory processes key to the pathogenesis to a number of varied neurological and neuropsychiatric conditions. We will combine the data from studies using PK and newer generation TSPO ligands to provide a detailed overview of how this research has led to a better understanding of the temporal and spatial relationships in microglial activation, neuroinflammation and disease manifestation. We will also discuss some of the limitations in TSPO PET imaging and suggestions for future progress in this field leading to a more personalised approach to treatment of neuroinflammation.

### Multiple Sclerosis

Multiple sclerosis (MS) is an autoimmune chronic condition characterised by migration of myelin-reactive T-cells, with subsequent microglial and astrocyte activation and recruitment of peripherally circulating macrophages causing demyelination and oligodendrocyte destruction within the CNS [16]. While MRI demonstration of lesions disseminated in time and space remains the diagnostic gold standard, TSPO PET imaging is a potentially promising tool not only in the diagnosis of MS, but in detecting the conversion from relapsing-remitting MS (RRMS) to secondary progressive MS (SPMS).

Studies have shown that activated microglia play a central role in sustaining chronic neuroinflammation in MS [17] and are found to have a specific and more diffuse distribution in SPMS when compared with RRMS [18, 19•]. In SPMS, greater mean TSPO radioligand uptake has been demonstrated throughout the white matter, as well as, specifically in the deep grey matter and the thalami of patients with SPMS [18, 19•, 20]. This increased cortical binding appears to correlate with disability, including impaired cognitive performance [18, 19•]. Further studies suggest that there is a positive correlation between TSPO ligand binding and disease severity [21, 22] and duration [20, 23]; however, the results are not consistent. In active disease, increased PK binding has been found to correspond to MRI gadolinium-defined active lesions, but not in chronic lesions [24, 25].This corresponds to similar findings by Oh and colleagues using the ^11^C-PBR28 ligand, which demonstrated that gadolinium-enhancing lesions had significantly higher binding compared with the contralateral white matter [23]. Additionally, PK binding in seemingly normal white matter has not only been found to have increased density in RRMS and SPMS patients compared with healthy controls, but it also appears to correlate with the degree of observed cerebral atrophy [26].

In clinically isolated syndrome (CIS), PK binding has been shown to have a potentially prognostic role. Gianetti and colleagues demonstrated that in patients with CIS who went on to develop MS within 2 years had higher PK binding on their baseline scans [22]. It has also been demonstrated that TSPO PET can be utilised to predict the development of MRI active lesions, suggesting microglial activation may play a pivotal role in MS lesion formation [23].

In general, PET imaging using second-generation TSPO tracers has been less successful in yielding results useful to the understanding of MS [27–29]. However, not all of the studies considered the implications of the rs6971 polymorphism in their analysis [28]. When this is taken into consideration, a study using the ^18^F-PBR111 radioligand revealed increased total distribution volume (V_T_, i.e. the ratio of radioligand concentration in the tissue target region to that in blood plasma at equilibrium) in MS patients compared with controls. There results also suggested an association between TSPO PET white matter signal and disease severity [21]. Similarly, Singhal and colleagues using the ^18^F-PBR06 ligand demonstrated its utility in assessing TSBO binding in MS [30, 31•]. They showed a significant correlation between microglial activation in deep grey matter, cerebellar white matter and white matter lesions associated with neurological disability and cerebral atrophy.

Finally, animal models have demonstrated that following treatment with immunosuppressive drugs, such as fingolimod, TSPO radiotracer uptake is reduced [32]. Similarly, clinical studies on patients with MS, published by Ratchford and colleagues, demonstrated that PK binding potential per unit volume was significantly decreased throughout the brain following treatment with glatiramer acetate after 1 year [33]. Potentially, therefore, TSPO PET could be used as a non-invasive biomarker to determine and monitor the efficacy of immunosuppressive therapies on MS disease activity.

### Stroke

Stroke researchers have utilised TSPO PET imaging to understand the role and time course of neuroinflammation following acute cerebral infarction. We know that following acute cerebral hypoxia, there is a significant increase in TSPO expression, most notably in astrocytes and microglia [34], with the inflammatory response divided into an initial release of pro-inflammatory mediators followed by a later neuroprotective phase distinguished by the release of anti-inflammatory mediators [35]. However, contradictory results [36] to this model suggest a much more complex and heterogeneously dynamic process making the development of immunomodulatory therapies problematic. Further complicating this, is the observation that neuroinflammation is not localised to the immediate surroundings of the infarct, but is also observed in remote brain regions that have fibre tract connections with the acutely affected area [37]. Activated microglia around areas of acute infarction exhibit distinct immunohistochemical properties compared with microglia distant from the lesion in the chronic stages following stroke [38]. Studies have demonstrated that areas remote to the infarcted area show evidence of extensive phagocytosis and iron deposition, compared with the relative resolution of neuroinflammation at the lesional site several months post-event [39, 40]. Such observations emphasise the need for reliable imaging biomarkers targeting neuroinflammation.

Studies using PK PET complement the immunohistochemical findings for the acute and chronic stages of stroke. Within a few days of the insult, increased PK binding is observed, with activated microglia present in the peri-infarct zone progressing to the ischemic zone a few days later [41]. The activation typically peaks approximately 1 week following the stroke, and thereafter it decreases in the acute region [42]. However, as with the immunohistochemistry, repetitive PK studies demonstrate marked neurodegeneration at sites remote from the lesion site as time post-event elapses. Evidence for this comes from a number of studies [37, 39, 40, 43] and is felt to represent Wallerian degeneration along connected pathways between different anatomical areas [44]. Thiel and colleagues described the temporal dynamics of locally activated microglia and their relationship to pyramidal tract damage in patients with subcortical stroke. They found that microglial activity at the infarct site decreases with recovery, with uptake ratios not significantly different to controls after 6 months, while activity persists in the brainstem along the affected pyramidal tract [37]. Similarly, Walberer and colleagues investigated neuroinflammation in the chronic stage of embolic stroke using a rat model and the PK ligand. The authors demonstrated that neuroinflammation all but resolved at the lesion site at 7 months, but with microglia activation detected at sites remote from the primarily infarcted regions, including the ipsilateral thalamus [39].

A potential limitation with the PK ligand regards its ability to differentiate between the infract and the peri-infarct region, in part due to low signal-to-noise ratio and non-specific binding. A number of comparative studies have aimed to improve the diagnostic power of TSPO PET using newer ligands, with differing degrees of success. Guylas and colleagues using ^11^C-vinpocentine measured regional changes of TSPO in the brain of nine ischemic stroke patients at nine different points between 1 and 14 weeks after the event [41]. At 1 week post-stroke, they observed an increased ^11^C-vinpocentine uptake in both the ischemic core and the peri-infract zone, with microglial activation seemingly more intense in the peri-infarct area. This increased uptake then decreased steadily with post-stroke time. Unfortunately, however, the authors noted that ^11^C-vinpocentine demonstrated low affinity for TSPO and in a further study, directly comparing ^11^C-vinpocentine with PK, they found that although ^11^C-vinpocentine had a greater affinity for TSPO than PK, the differences were not significant [45].

More promise has been seen with the ^18^F-DPA-714 and ^11^C-DPA-713 ligands. In one such study, nine patients underwent PET imaging using the ^18^F-DPA-714 radio-ligand between 8 and 18 days after stroke [46]. The authors demonstrated that observed increases in ligand uptake co-localised with the infarcted tissue and extension beyond the region corresponding to the damage in the blood-brain barrier. This suggests that ^18^F-DPA-714 may be useful in assessing the extent of neuroinflammation associated with acute stroke, although it must be noted that no correlation was identified between tracer uptake and infarct volume. A recently published study by Chaney and colleagues, where mice were subject to middle cerebral artery occlusion or sham surgery, compared the two second-generation ligands ^11^C-DPA-713 and ^18^F-GE-180 [47•]. While both ligands were able to detect neuroinflammation at acute and chronic time points following the ischaemic event, ^11^C-DPA-713 was found to be more sensitive in reflecting the extent of glial cell activation and allowed earlier detection compared with ^18^F-GE-180 [47•]. Such findings support TSPO as a useful biomarker of neuroinflammation in distinguishing the sub-acute and chronic phases post-stroke.

Both PK and newer TSPO ligands have been key in elucidating how changes in TSPO expression, both spatially and temporally, relate to the pathogenesis of stroke and highlight a conceivable role for novel anti-inflammatory substances in the long-term management of stroke. Furthermore, TSPO ligands, such as ^18^F-DPA-714 and ^11^C-DPA-713, have a potential role in tracking in vivo microglial activation, which may allow predictions regarding individual functional recovery and assessing the utility of future therapeutic strategies [46, 48].

### Traumatic Brain Injury

Traumatic brain injury (TBI) is a disease with many different symptoms including cognitive, emotional and physical impairments. In general, the majority of TBIs are single events; however, repeated injuries among affected people are related to the development of chronic traumatic encephalopathy (CTE), even though the pathology of CTE can also be seen after single TBI [49•]. Mechanisms responsible for these disabilities can be divided into primary and secondary injuries. While the primary injury is the initial biomechanical trauma [50], a process involving neuronal, axonal and vascular damage induced by the kinetic energy, this induces a cascade of secondary processes leading to excitotoxicity, necrosis, apoptosis, autophagy and free radical formation [51]. Hence, TBI is considered to be a chronic disease [52], implicating that the pathophysiological and inflammatory processes in the brain take place at different times after the injury, where some are beneficial to recovery and others are generated by the injury and exaggerate the primary damage [53, 54].

Brain injury can trigger neurodegeneration and is considered a major riskfactor for the development of dementia, as highlighted in one study b y the accumulation of amyloid-β plaques in around 30% of post-mortem brain tissue collected from TBI patients [55]. Interestingly, PET imaging studies in TBI patients revealed a similar distribution of amyloid plaques as in patients with Alzheimer’s disease [56]. Further, TBI has been associated with Parkinson’s disease [57] and various psychiatric disorders including increased risk of suicide, and overall increase in mortality [58].

Areas of activated microglia often coincide with observed neuronal degeneration and axonal abnormality [59, 60]. Studies in animal models [61] and in humans [62, 63] have detected microglial activation occurring early after TBI, but then persisting for years, detectable both in vivo and post-mortem. A study by Ramlackhansingh and colleagues using PK in patients 11 months to 17 years post-TBI found binding to be significantly increased in the thalami, occipital cortices, putamen and posterior limb of the internal capsules, but with no increase at the original site of injury [62]. High PK binding in the thalamus corresponded to worse cognitive outcomes and increased microglial activation was identified up to 17 years following head injury, supporting the premise that TBI triggers a chronic neuroinflammatory response [62].

These findings have been replicated in further studies, with one group showing that PK binding was again increased in subcortical regions remote from acute injury site [64] and in models of experimental TBI [53]. The prominence of microglial activation in the subcortical structures may reflect their dense connectivity and suggests that microglia behave differently locally to site of injury from those at remotely connected structures. It is likely that this reflects a slowly progressive process within damaged white matter similar to what is observed in stroke [49•].

Recent investigations using ^11^C-DPA-713, a second-generation TSPO tracer, in older former National Football League (NFL) players revealed significantly higher TSPO PET signal in the right amygdala and bilateral supramarginal gyri of the players compared with controls [65]. Further, Coughlin and colleagues [66•] recently reported increased TSPO binding in predominantly medial temporal lobe regions and subtle evidence of white matter damage on diffusion MRI in a group of 14 active or recently retired NFL players with a history of concussions. Previous PK studies in both TBI and stroke have demonstrated that ligand binding correlated with the extent of white matter damage and axonal injury may be an important factor in causing persistent microglial activation leading to progressive degeneration [49•, 62]. Animal models of closed TBI using ^18^F-DAP-714 have demonstrated that uptake correlated with trauma severity, metabolic deficits and the degree of microglial activation [67•].

The clinical significance of persistent microglial activation in TBI is unclear, however one can deduce that the balance of activation states/polarisation varies at different points [49•]. The relevance of microglia in the general pathophysiological response to TBI is potentially therapeutically relevant, and there is interest in targeting microglial pathways and immune modulation to prevent neurodegeneration [68–70]. Longitudinal studies are required to clarify the functional significance of microglial activation in remote parts of the brain and determine the reliability of TSPO ligands as markers for severity and progression in TBI [62, 71].

### Neuroinflammation in Human Immunodeficiency Virus (HIV) Infection

Microglia activation appears to play a role in the development of cognitive impairment and dementia associated with HIV infection. In a pilot study, five healthy volunteers and 10 HIV-positive patients, with and without HIV-associated dementia (HAD), underwent PET with the PK ligand [72]. As a group, the HIV-positive patients overall showed significantly higher tracer binding than controls in five brain regions, and similar results have been observed using newer TSPO radio-ligands, such as ^11^C-PBR28 [73•].

Patients with HAD did not show any significant difference in PK-binding compared with HIV-positive, non-demented patients. However, while non-demented HIV-positive patients did not show any significantly increased binding compared with controls, HAD patients demonstrated significantly higher PK-binding than controls in five out of eight brain regions, supporting a possible role for microglial activation in HAD [72]. In contrast to this, another PET study using the PK radio-ligand in 12 HIV-infected patients with minor neurocognitive impairment and 5 controls found no increase in PK-binding in the HIV-infected patients compared with controls in any of the investigated brain regions [74]. The authors concluded that PK-binding PET might be insensitive to the degree of macrophage activation in HIV-associated minor neurocognitive impairment or, alternatively macrophage activation is not implicated in this condition. A third alternative, however, is that the ROI approach used in this study is not sensitive enough to detect subtle and localised increases in activated microglia in cortical areas. In fact, using voxel-by-voxel analysis, Garvey and colleagues detected the presence of activated microglia in several focal cortical areas in asymptomatic HIV-infected patients [75]. Additionally, increased PK-binding in the anterior cingulate, corpus callosum and posterior cingulate correlated with poorer executive performance.

TSPO binding using the ^11^C-PBR28 ligand found global increases in TSPO expression, with significant regional increases in the occipital lobe, parietal lobe and globus pallidus in patients who were HIV-positive when compared with controls. This same study found that increased TSPO binding in the amygdala, thalamus and hippocampus correlated with poorer global cognitive performance, particularly with verbal and visual memory [73•]. It remains to be established whether detection of activated microglia in HIV-positive patients is a predictor of future neurocognitive decline.

### Neuropsychiatric Disease

TSPO PET-studies provide evidence that impaired regulation of microglia contributes to both neurobehavioural and neuropsychiatric disorders. In schizophrenia, for example, one of the most consistent genetic associations relating to this disorder concerns the major histocompatibility complex, and therefore the innate immune system [13•]. The pathology of schizophrenia has been associated with neuroinflammation, and the relevance of microglial activation can be observed in the inhibitory effects of both typical and atypical anti-psychotics on activated microglia [76]. Two small studies utilising PK have demonstrated increased TSPO signal in the grey matter, hippocampus and temporal cortex of patients with schizophrenia [77, 78]. Unfortunately, later studies using larger cohorts and new TSPO radioligands have been unable to replicate these results in patients with early-stage psychosis or schizophrenia when compared with healthy controls [79–82]. In one such study, while Takano and colleagues demonstrated a positive correlation between positive symptom scores, disease duration and ^11^C-DAA1106 binding, there was no significant difference in total binding in cortical areas when compared with controls [80]. Another recent study detected no difference in ^18^F-FEPPA binding between 19 untreated patients with first-episode psychosis compared with 20 controls [83].

However, more promising insight arises from a study utilising the ^11^C-PBR28 radio-ligand. The investigators found that in schizophrenic patients the total cortical grey matter volume was significantly lower when compared with healthy controls [84•]. Patients were genotyped for the rs6971 polymorphism. This corresponded to a negative correlation between TSPO signal and the total cortical grey matter volume. While these findings suggest that in schizophrenia microglial activation is related to altered cortical volume, longitudinal studies will be required to determine the exact relationship between microglial activation and cortical grey matter loss, and whether anti-psychotic treatment has any effect on changes in brain volume observed in these patients.

Investigations in depression have also not yielded conclusive results, with one such study concluding that there was no statistical difference between patients with mild to moderate depression and healthy controls using ^11^C-PBR28 PET [85]. However, studies are emerging that challenge this in more severely affected patients and demonstrating the effect of treatment on neuroinflammation. Setiawan and colleagues using the ^18^F-FEPPA ligand found slightly elevated V_T_ values in the anterior cingulate cortex of 20 patients with major depressive episode (MDE) compared with 20 controls [86]. This area is implicated in the control of emotional behaviour, and this finding represents the first evidence of significant increase in brain TSPO density in vivo during MDE [86]. This finding has recently been replicated by Richards and colleagues using the ^11^C-PBR28 TSPO radio-ligand in patients with MDE [87•]. They identified a trend towards increased ^11^C-PBR28 binding in patients with MDE compared with healthy controls, and post hoc analysis further demonstrated that this abnormality was significant in the unmedicated MDE patients. Similar findings have also been published regarding PK TSPO binding, which have found that binding was significantly elevated in the anterior cingulate and insula in patients with co-existent MDE and suicidal ideation [88].

In further work, Setiawan and colleagues again demonstrated using 18F-FEPPA PET that TSPO V_T_ is increased in patients with more advanced MDE who have had longer periods without anti-depressant treatment compared with those who have had shorter periods without medication [89••]. They further found that TSPO binding was between 29 and 33% greater in the prefrontal cortex, anterior cingulate cortex and insula in patients with longer disease duration [89••]. These results are strongly suggestive of a different illness phase, which has implications for staging MDE, and the authors also found that the yearly increase in microglial activation they observed in untreated patients (14–18% per decade) disappeared when anti-depressants were initiated [89••]. This is not surprising knowing that serotonin reuptake inhibitors are shown to inhibit microglial activation [90, 91]. These results, therefore, support the theory that major depression is due to neuropathological progression secondary to chronic microglial activation and are consistent with the observed clinical transition from infrequent solitary episodes, with inter-episode recovery, towards more persistent disease [89••].

A further recent study demonstrated that when ^18^F-FEPPA PET results were compared before and after treatment with cognitive behavioural therapy in patients with MDE; *V*_T_ values were significantly reduced during the treatment period [92]. Interestingly, reduction in *V*_T_ values was not significant in the cohort who received supportive psychotherapy. However there was a correlation observed in both treatment groups between reduction in TSPO *V*_T_, consistent in the hippocampus and amelioration of depressive symptoms [92]. Interestingly, studies comparing patients with bipolar disorder to healthy controls using PK also found significantly increased tracer binding in the hippocampus [93, 94]. Thus, highlighting that hippocampal TSPO overexpression appears to be a shared characteristic between distinct psychiatric disorders.

Certainly, there is an accumulating body of evidence associating the pathophysiology of neuropsychiatric conditions with dysregulation of the immune response. However, the heterogeneity within study populations, as well as relatively small sample sizes, makes replication of studies in mental health research more difficult. Considering the PK and ^11^C-PBR28 findings in schizophrenia, the patients recruited had an established diagnosis, and in a similar pattern to MDE, the results may indicate that the aberrant immune response is representative of the pathophysiology in the chronic phase of illness with progressive change occurring at the glial level. This warrants clarification with further studies.

### Limitations of TSPO PET Imaging

There are a number of limitations concerning the utility of TSPO as a PET microglial biomarker. These include low brain density, expression by cells other than microglia, similar expression of activated microglia in with M1 and M2 states and the incidence of the aforementioned genetic polymorphism. Certainly, it is not clear whether increased or decreased TSPO binding reflects a particular microglial phenotype, meaning that TSPO PET does not differentiate between a specific functional role, i.e. neurotoxic (M1) versus neuroprotective (M2) [1••]. It is, therefore, more accurate to state that upregulation of TSPO depicts the broader multicellular neuroinflammatory reaction, rather than simply reflecting microglia activation [95•]. This statement is supported by studies showing that TSPO expression in both microglia and astrocytes appears to be temporally distinct, depending on the stage of disease progression as much as on the disease itself. For example, in some rat models of active MS, increased TSPO expression is localised to microglial cells [25], in contrast to post-mortem animal and human data demonstrating involvement of both astrocytes and microglia [96, 97]. The presumption is that initial microglial activation is followed by delayed but prolonged astrocytic activation.

Another important consideration is the estimation of specific TSPO binding. Typically, kinetic modelling is the standard for PET quantification; however, this can be challenging with TSPO radioligands [1••]. Studies using both PK and second-generation ligands have reported significant between-subject variability in *V*_T_ when using the standard approach of arterial sampling for kinetic modelling [95•]. For PK, this is at least in part due to high binding to plasma proteins altered in pro-inflammatory states [98], and this may also be the case for second-generation ligands, albeit unconfirmed. The result is considerable variability in *V*_T_ within and across studies, and high vascular TSPO binding is further implicated to substantially affect outcome estimation [13•]. Limitations regarding the different generations of radioligands are summarised in Table 1.

**Table 1:** Limitations of TSPO radiotracers

First generation TSPO [12, 99]	Second generation TSPO	Third generation TSPO
Short half-life	rs6971 polymorphism	Challenge of absolute quantification and kinetic modelling
Poor signal-to-noise ratio	No differentiation between M1 and M2	No differentiation between M1 and M2
Non-specific binding	Slow accumulation of radio metabolites resulting in inaccurate estimations of TSPO [100, 101]	Limited data available
	Slow kinetic behavior necessitating longer scanning time [101]	

The challenge of absolute quantification has lead researchers to find alternative methods to an arterial input function, including reference tissue modelling [95•]. However, unlike other ligands targeting neurotransmitter systems, there is not part of the brain parenchyma devoid of TSPO expression and so no true reference region exists [95•]. Nonetheless, different approaches have been sought. Considering PK, supervised clustering methods have enabled the identification of reference tissue [102]. While this is not applicable to second-generation ligands [81], a study using ^11^C-PBR28 in Alzheimer’s patients was able to estimate binding using a cerebellar pseudo-reference region, demonstrating less variability compared with estimation using plasma activity [103]. Non-invasive techniques pose attractive alternatives to the less reliable arterial sampling in TSPO studies, but validation using such techniques is paramount concerning the ligand and population being studied.

### Future Directions

Although the definitive functional role of TSPO continues to elude us, techniques such as X-ray crystallography have revealed its pentameric 3D structure, including selective bindings sites [104] [105]. These binding sites could prove novel therapeutic targets and models of CNS injury appear to show that PK has a neuroprotective effect with a reduction in reactive microglia and astrocytes [106, 107]. However, without steadfast knowledge on how the upregulation of TSPO influences the immune reaction, development of affective treatments will remain limited. For example, there is an evidence to suggest that TSPO may play a role as a negative regulator of inflammatory signalling in macrophages [108]. This supports the argument that increased TSPO binding, in certain circumstances, in fact, reflects a protective response rather than a pro-inflammatory, tissue-damaging phenotype, and microglial activation could be a normal response to independent pathological processes. This has significant therapeutic implications when considering TSPO-dependent agonistic versus antagonistic strategies, and exploration for alternative PET-based ligands (Table.2) targeting sensitive markers of glial activation [1••], as well as the immune response, will be crucial to progress in this field.

**Table 2:** Examples of potential alternative molecular targets for radioligands to facilitate CNS PET imaging of microglial activation. Both current and proposed molecular targets are outlined

Molecular target	Cellular localisation	Cell type expression	Function	M1/M2 differentiation	Current application or proposed
COX	Cytoplasmic enzyme	Microglia, neurones	Synthesis of prostaglandins	No data yet available	Current—murine models
P2X7R	Cation-permeable ion channel receptor	Microglia, astrocytes, macrophages, Schwann cells	Activates proinflammatory cytokines (IL-1β) and ROS release	Potentially is specific for the M1 phenotype	Current—murine model
CB2R	G protein-coupled receptor	Microglia, astrocytes, microvascular endothelial cells	Inhibits release of pro-inflammatory cytokines (IL-1, TNF-α) and activates release of anti-inflammatory cytokines (IL-4, IL-10)	No data yet available	Current—both murine and human studies
A2AR	G protein-coupled receptor	Microglia, astrocytes, neurones	Anti-inflammatory effects	No data yet available	Current—human studies
β-Glucuronidase	Lysosomal enzyme	Microglia, astrocytes, neurones	Anti-inflammatory effects	No data yet available	Current—murine model
MMPs	Immature cytoplasmic enzymes which are activated extracellularly	Microglia, astrocytes, neurones, oliodendrocytes	CNS development, e.g. neurogenesis and axonal guidance	No data yet available	Current—murine model
α4β2 nAChR	Pentameric nicotinic receptor	Microglia, neurones	Anti-inflammatory effects	No data yet available	Current—murine model
iNOS	Cytoplasmic enzyme	Microglia, astrocytes, macrophages	Nitric oxide production by immune cells	Potentially specific for the M1 phenotype	Proposed
IDO-1	Cytoplasmic enzyme	Microglia, neurones	Tryptophan catalisation	No data yet available	Proposed
KMO	Cytoplasmic enzyme	Microglia, macrophages	Tryptophan catalisation	No data yet available	Proposed
FR-β	Surface receptor	Microglia	Captation and internalisation of folic acid	Potentially specific for the M2 phenotype	Proposed
P2Y12R	Purinergic G protein-coupled receptor	Microglia	Role in platelet aggregation	Potentially specific for the M2 phenotype	Proposed

*COX* cyclooxygenase; *P2X7R* purinergic receptor two ion channel receptor; *CB2R* cannabinoid receptor type 2; *A2AR* adenosine receptor 2A; *MMP* Matrix metalloproteinases; *nAChR* nicotinic acetylcholine receptor; *iNOS* inducible nitric oxide synthase; *IDO-1* indoleamine 2,3-dioxygenase 1; *KMO* kynurenine-3-monooxygenase; *FR-β* folate receptor β; *P2Y12* purinergic ion channel Y12; *IL* interleukin; *ROS* reactive oxygen species; *TNF-α* tumour necrosis factor-α; *M1* pro-inflammatory activated microglia; *M2*: anti-inflammatory microglia

## Conclusion

It is increasingly apparent that neuroinflammation is implicated in a diverse range of neurological and neuropsychiatric conditions. Despite the discussed limitations, it should be apparent that TSPO has emerged as an important neuroinflammatory biomarker for disease monitoring in vivo and contributing to our understanding of the manifestation chronic disease states.

While the development of novel techniques should be encouraged, neuroimaging researchers should continue to build on existing methods to drive innovation forward and overcome the inherent shortcomings in TSPO PET. Through the accurate depiction of the pathophysiological processes, we can not only improve diagnostic techniques, but develop new and effective immunomodulatory treatments, with the exciting potential of future neurorestorative therapies.
